# Dietary Adherence, Orthorexia Nervosa Risk, Physical Activity Levels, and Body Image Satisfaction in Patients with Neuroendocrine Tumors: A Cross-Sectional Study

**DOI:** 10.3390/nu18142254

**Published:** 2026-07-10

**Authors:** Giovanni Guarascio, Francesca Greco, Edoardo Mocini, Arianna Gagliardi, Gian Pietro Emerenziani, Elisa Giannetta, Maria Grazia Tarsitano

**Affiliations:** 1 Department of Experimental and Clinical Medicine, University “Magna Græcia” of Catanzaro, 88100 Catanzaro, Italy; giovanni.guarascio002@studenti.unicz.it (G.G.); emerenziani@unicz.it (G.P.E.); 2 Department of Mathematics and Computer Science, University of Calabria, 87036 Rende, Italy; francesca.greco.demacs@unical.it; 3 Department of Theoretical and Applied Sciences, eCampus University, 22060 Novedrate, Italy; edoardo.mocini@uniecampus.it; 4 Department of Experimental Medicine, Sapienza University of Rome, 00161 Rome, Italy; arianna.gagliardi@uniroma1.it; 5 Department of Human Science and Promotion of Quality of Life, San Raffaele Open University of Rome, 00166 Rome, Italy; mariagrazia.tarsitano@uniroma5.it

**Keywords:** neuroendocrine tumors, orthorexia nervosa, mediterranean diet, physical activity, body image, nutritional counselling

## Abstract

**Background/Objectives**: The global incidence of neuroendocrine tumors (NETs) is steadily rising. While modifiable lifestyle factors impact oncological outcomes, the interplay between dietary habits, orthorexia nervosa (ON) tendencies, physical activity levels (PAL), and body image in NET survivors remains poorly understood. **Methods**: A cross-sectional study was conducted on a convenience sample of 61 patients with NETs. Validated evaluation tools included the PREDIMED questionnaire for Mediterranean Diet (MD) adherence, the GPAQ for PAL, and the ORTO-15 questionnaire for ON risk. Body image satisfaction and comprehensive clinical features were also assessed. **Results**: Most participants (75.4%) demonstrated moderate MD adherence. Body image dissatisfaction (32.8%) was significantly associated with a higher BMI (28.86 vs. 24.93 kg/m^2^, *p* < 0.01) and reduced PAL (660.0 vs. 1680.0 MET-min/week, *p* = 0.021, pFDR = 0.042). Notably, moderate MD adherence was linked to a higher orthorexic risk (corresponding to lower ORTO-15 scores) compared to low adherence (*p* = 0.032, pFDR = 0.078). Somatostatin analog therapy showed no detrimental impact on behavioral parameters (*p* > 0.05). **Conclusions**: These findings highlight specific vulnerabilities in NET patients: body image dissatisfaction correlates with higher BMI and lower physical activity, while moderate Mediterranean Diet adherence associates with orthorexic risk, which is localized and was not independent in multivariate analysis. Targeted nutritional and physical activity interventions should be integrated into multidisciplinary NET care.

## 1. Introduction

Neuroendocrine Neoplasms (NENs) comprise a heterogeneous group of tumors arising from neuroendocrine cells and may develop in virtually any organ, although 70% of NENs affect the digestive system, the gastroenteropancreatic (GEP) tissue [1,2]. Furthermore, although traditionally considered rare, the global incidence of these malignancies has steadily and significantly increased over the past few decades [3,4]. According to the current World Health Organization (WHO) classification [4,5], NENs are broadly divided into well-differentiated neuroendocrine tumors (NETs) and poorly differentiated neuroendocrine carcinomas (NECs), a distinction based on morphology, differentiation, and proliferative activity. NETs are graded as G1, G2, or G3 on the basis of mitotic count and/or Ki-67 proliferation index, whereas NECs are by definition high-grade neoplasms and are further classified as small-cell or large-cell carcinoma [4,5]. This updated framework reflects the biological and clinical heterogeneity of NENs and improves diagnostic accuracy, prognostic stratification, and therapeutic planning [2,4]. In recent years, the incidence and prevalence of NENs have increased worldwide, likely due to a combination of improved detection, greater awareness, and true epidemiologic changes [2,6]. Given this growing burden, understanding modifiable lifestyle factors may be relevant not only for cancer prevention but also for patient management and survivorship [7]. In this context, dietary patterns, nutrition status and physical activity have attracted increasing attention because they may influence overall health, treatment tolerance, and quality of life in patients with cancer [7,8,9]. Our study focuses on two lifestyle factors that may influence NENs, dietary habits and physical activity, with particular emphasis on adherence to the Mediterranean diet (MD) and orthorexia nervosa (ON). Despite growing clinical interest, it is important to note that ON is not yet formally recognized as a distinct eating disorder in the Diagnostic and Statistical Manual of Mental Disorders, Fifth Edition, Text Revision (DSM-5-TR) [10,11]. Its diagnostic validity remains highly debated due to symptomatic overlap with other psychiatric conditions, such as Anorexia Nervosa and Obsessive–Compulsive Disorder; nevertheless, this behavioral pattern has attracted significant research attention, highlighting the pressing need for standardized diagnostic criteria [10,11]. The MD has been associated with favourable cardiometabolic effects and may contribute to cancer prevention and improved outcomes through its anti-inflammatory and nutrient-dense profile [12]. Although dietary habits are crucial to a better prognosis and risk of recurrence [7], a cancer diagnosis can motivate people to change their eating habits [13]. There is evidence of a link between cancer and certain types of altered eating behavior, like ON, food cravings and food addiction [14,15,16]. Moreover, several studies highlight a correlation between body image dissatisfaction and altered eating behaviour in this population [17,18]. Given the importance of nutrition and the increasing number of cancer survivors, it is important to study their dietary habits and how these may affect survival and quality of life [19]. Due to the scarcity of evidence specifically addressing NENs, evaluating the interplay between MD adherence and ON behaviours in this setting is of particular interest. Physical activity is another key modifiable factor associated with multiple health benefits, including improved cardiovascular fitness, weight control, psychological well-being, and musculoskeletal health [20]. In oncology, exercise has been linked to better quality of life, reduced cancer-related fatigue during and after treatment, and lower risk of recurrence and mortality in several tumor types [21,22]. Evidence also suggests that higher levels of physical activity may be inversely associated with cancer incidence overall [22,23]. Accordingly, our study also examines the relationship between physical activity and NENs in this patient population. In summary, while the current literature highlights the influence of adherence to the MD [12], ON [14] and physical activity [21,22] on various types of cancer, there remains a notable lack of evidence specific to NENs. We hypothesized that NEN patients present a unique behavioral and psychological profile compared to other cancer survivors. Biologically, the hormonal hypersecretion characteristic of many functioning NENs (e.g., carcinoid syndrome) causes chronic gastrointestinal symptoms that are frequently exacerbated by food intake [9,24]. Psychosocially, the relatively prolonged survival times typical of NENs mean that patients must endure this symptom burden for years [3,25,26]. This continuous struggle may prompt patients to exert rigid, obsessive control over their diet in an attempt to manage their condition, potentially increasing their vulnerability to maladaptive eating behaviors such as ON. Given the unique characteristics of these tumors and their treatment challenges, it is essential to investigate how these three factors interact in this patient population. Therefore, our study aims to assess the relationships between physical activity, body image satisfaction, adherence to the MD and ON in patients with NENs.

## 2. Materials and Methods

### 2.1. Participants

In this cross-sectional study, a total of 61 patients with a confirmed diagnosis of NETs were recruited from the outpatient clinics of Policlinico Umberto I (Rome, Italy). Inclusion criteria were (a) histologically or cytologically confirmed diagnosis of NEN, (b) age ≥ 18 years, and (c) provision of written informed consent. Exclusion criteria were (a) presence of concomitant malignant diseases other than NETs, (b) incomplete clinical documentation, (c) acute medical conditions interfering with study assessments, and (d) refusal or inability to provide informed consent. Demographic information, medical history, and disease characteristics were extracted from the institutional electronic medical records. Ethical approval was granted by the local Ethics Committee (Local Ethics Committee of Lazio Area, approval no. 0390/2024, 24 April 2024).

### 2.2. Experimental Procedure

Data collection was conducted by two qualified research staff members from Policlinico Umberto I, who directly administered the surveys. During the initial interview, patients were asked to indicate their marital status (married, single, cohabiting, widowed, or separated) and their highest level of education (primary school, middle school, high school diploma, bachelor’s degree, or master’s degree). Additionally, participants were asked a single dichotomous question to assess their body image satisfaction (“Are you satisfied with your body image?”, categorized as Yes or No). After collecting sociodemographic information, the operators administered three validated questionnaires in a standardised order. First, the PREDIMED (PREvencion con Dieta MEDiterranea) questionnaire, a validated 14-item tool, was used to measure adherence to the MD [27]. Each response was scored as either 1 or 0, yielding a total score that was categorized into three adherence levels: low adherence (scores from 0 to 5), moderate adherence (scores from 6 to 9), and high adherence (scores of 10 or greater) [27]. Physical activity levels (PAL) were assessed using the Global Physical Activity Questionnaire (GPAQ) [28]. This questionnaire assesses physical activity by examining three distinct domains: work-related, transport-related, and exercise activities. Within each category, participants specified both the weekly frequency (days per week) and the daily duration (minutes per day) dedicated to moderate- and vigorous-intensity physical efforts. Total weekly energy expenditure was then determined by multiplying the daily minutes, weekly days, and the corresponding Metabolic Equivalent (MET) coefficients, specifically 4.0 METs for moderate-intensity and 8.0 METs for vigorous-intensity tasks. The final values were expressed as MET-minutes per week (MET-min/week) [28]. Finally, ON tendencies were evaluated using the ORTO-15 questionnaire [29]. This widely accepted 15-item tool assesses a participant’s obsessive attitude toward healthy eating, focusing on the selection, preparation, and consumption of food. Each item is scored on a 4-point Likert scale (ranging from 1 to 4), where a score of 1 reflects more severe orthorexic behaviors and a score of 4 indicates normal eating habits. The total score ranges from 15 to 60. A cut-off threshold of less than 35 was utilized to identify individuals at risk of ON [30,31,32]. In our specific sample, the internal consistency of the questionnaire yielded a Cronbach’s alpha of 0.643, confirming a moderate reliability that is consistent with the previous literature [10,31]. All interviews were conducted following a structured protocol to ensure consistency among assessors, and responses were recorded in a secure electronic database for subsequent analysis.

### 2.3. Statistical Analysis

The normality of data distribution for all continuous variables was assessed using the Shapiro–Wilk test. For this study, the primary outcomes of interest were defined as adherence to the Mediterranean diet (PREDIMED score), orthorexia nervosa risk (ORTO-15 score), and body image satisfaction, while physical activity levels (PAL) and Body Mass Index (BMI) were designated as secondary outcomes. Accordingly, normally distributed continuous data (age, body mass, height, Body Mass Index (BMI) and ORTO-15) are presented as means and standard deviations (SD). Conversely, non-normally distributed continuous variables (duration of NET, age at diagnosis, PREDIMED score and PAL) are reported as medians and interquartile ranges (IQR). Categorical parameters are expressed as absolute frequencies and percentages. Associations between categorical variables (e.g., tumor grading, tumor localization, and pharmacological therapy) were evaluated using the Chi-square test of independence. To further quantify the strength of the significant associations in 2 × 2 contingency tables, the Odds Ratio (OR) with its corresponding 95% Confidence Interval was calculated. For between-group comparisons involving two independent categories (like gender, grading, body image satisfaction, and tumor localization), the independent-samples *t*-test was employed for normally distributed variables, whereas the Mann–Whitney U test was used for non-parametric data. Because there was only a single G3 patient, we decided to exclude this case from the comparative analysis and performed independent-samples *t*-tests and Mann–Whitney U tests exclusively between the G1 and G2 groups. The single patient diagnosed with a G3 tumor was excluded from comparative analyses. Retaining a group with a sample size of one (*n* = 1) prevents the estimation of variance and strictly violates the mathematical assumptions of between-group statistical tests; consequently, grading-based comparisons were performed exclusively between the G1 and G2 groups. To compare continuous variables across three or more independent groups (e.g., marital status, educational level and PREDIMED adherence categories), a one-way analysis of variance (ANOVA) was conducted. The assumption of homogeneity of variances was preliminarily verified using Levene’s test. When the ANOVA yielded a statistically significant main effect, Tukey’s Honestly Significant Difference (HSD) post hoc test was applied to perform pairwise comparisons. To assess the clinical magnitude and practical relevance of the findings, effect size measures were calculated and reported: Cohen’s d for independent samples *t*-tests (and post hoc pairwise comparisons), eta-squared (η^2^) for ANOVA, and rank-biserial correlation (r) for Mann–Whitney U tests. To control the family-wise error rate due to multiple comparisons, the Benjamini–Hochberg False Discovery Rate (FDR) procedure was applied to the primary univariate analyses, and adjusted *p*-values (p_FDR) are reported alongside raw *p*-values where appropriate. For non-normally distributed variables, the non-parametric Kruskal–Wallis H test was utilized. Additionally, bivariate correlation analyses were conducted to explore potential linear relationships among clinical, anthropometric, and psychometric/dietary parameters. Pearson’s correlation coefficient was used for normally distributed data, while Spearman’s rank correlation coefficient was utilized for non-parametric variables. Missing data were handled using a pairwise deletion approach to maximize the retention of available data for each specific statistical test. All statistical procedures were performed using jamovi software (Version 2.3; jamovi project, 2024), with the threshold for statistical significance set a priori at *p* < 0.05. To identify independent factors associated with body image satisfaction, dietary adherence, and orthorexia risk, multivariate regression analyses were constructed. Specifically, a multivariate logistic regression model was used to evaluate predictors of body image dissatisfaction (0 = Satisfied, 1 = Dissatisfied) in the overall cohort, adjusting for age, gender, and tumor location (GEP vs. non-GEP). Predictors included BMI and physical activity levels (PAL, scaled per 1000 MET-min/week). An additional model was run in the graded subgroup (*n* = 40) to incorporate tumor grade (G1 vs. G2) as a covariate (excluding the GEP variable due to near-zero variance). Multiple linear regression models were also constructed to examine independent associations of ORTO-15 and PREDIMED scores as continuous variables, adjusting for clinical and demographic characteristics. Multicollinearity was assessed using the Condition Number.

Given the rarity of NETs and the exploratory nature of this study, a formal a priori power calculation was not performed. The sample of 61 patients represents a consecutive convenience sample recruited over the study period and reflects the real-world availability of eligible patients at our center. To justify this sample size retrospectively, a sensitivity power analysis was performed for bivariate correlations on the overall cohort. Given our sample size of 61 patients and a significance level of α = 0.05 (two-tailed), the study was powered to detect a minimum correlation coefficient of Cohen’s r = 0.348 with 80% power (1 − β = 0.80). This indicates that the study was adequately powered to identify moderate-to-strong associations among the clinical, anthropometric, and behavioral variables.

## 3. Results

A total of 61 individuals with NET (females *n* = 31, males *n* = 30) were enrolled. All participants completed the full experimental protocol. The characteristics of participants are presented in Table 1.

**Figure 1:**
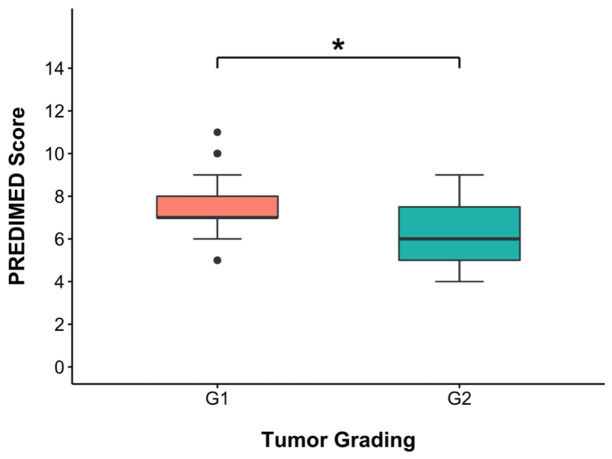
Box plot of PREDIMED score in patients with G1 versus G2 tumors. A non-parametric Mann–Whitney U test indicated a statistically significant between-group difference (*p* = 0.043). The box spans the interquartile range (25th–75th percentile) with the median shown as a horizontal line. Whiskers represent minimum and maximum values, while individual black dots represent outlier data points. * represents statistically significant difference (*p* < 0.05).

Among the participants, the majority were married (65.6%, *n* = 40). The remaining patients were single (14.8%, *n* = 9), separated (8.2%, *n* = 5), widowed (6.6%, *n* = 4), or cohabiting (4.9%, *n* = 3). Regarding educational level, 8.2% (*n* = 5) had completed primary education, and 26.2% (*n* = 16) had a middle school education. The most common educational qualification was a high school diploma (42.6%, *n* = 26). At the university level, 3.3% (*n* = 2) of the participants had earned a bachelor’s degree, while 19.7% (*n* = 12) held a master’s degree. Within the sample, the majority of the cohort (63.9%, *n* = 39) was diagnosed with gastroenteropancreatic neuroendocrine tumors (GEP-NETs), whereas the remaining 36.1% (*n* = 22) presented with non-GEP NETs. Tumor grading was available for a total of 41 patients, encompassing all 39 GEP-NETs and 2 non-GEP NETs. Within this graded subgroup, 61.0% (*n* = 25) were classified as G1, 36.6% (*n* = 15) as G2, and 2.4% (*n* = 1) as G3. In terms of pharmacological management, data regarding somatostatin analog (SSA) therapy was available for 57 participants, with 45.6% (*n* = 26) of them currently receiving the treatment. A Chi-squared test revealed a statistically significant association between tumor site classification (GEP-NET vs. non-GEP NET) and pharmacological management (x^2^ = 5.28, *p* = 0.022, pFDR = 0.031). Specifically, a higher proportion of patients diagnosed with GEP-NETs were currently receiving SSA therapy compared to those with non-GEP NETs (56.8%, *n* = 21 vs. 25.0%, *n* = 5). OR = 3.94, 95% Confidence Interval = 1.18 to 13.12. To evaluate the association between tumor grading and the administration of SSA therapy within the GEP-NETs group, a chi-square test of independence was performed. This analysis revealed a statistically significant relationship (x^2^ = 6.96, *p* = 0.031, pFDR = 0.031) OR = 6.22, 95% CI = 1.36–28.37. In particular, patients with G2 tumors were more frequently treated with SSA (80.0%, *n* = 12/15) compared to those with G1 tumors (39.1%, *n* = 9/23). Furthermore, when comparing continuous variables based on tumor grading, a non-parametric Mann–Whitney U test indicated a statistically significant difference in the PREDIMED score between the two groups. Specifically, patients classified as G1 exhibited a significantly higher adherence to the MD compared to those classified as G2 [7.0 (IQR 1.0) vs. 6.0 (IQR 2.5), U = 259, *p* = 0.043, pFDR = 0.086, r = 0.38] (Figure 1).

**Figure 2:**
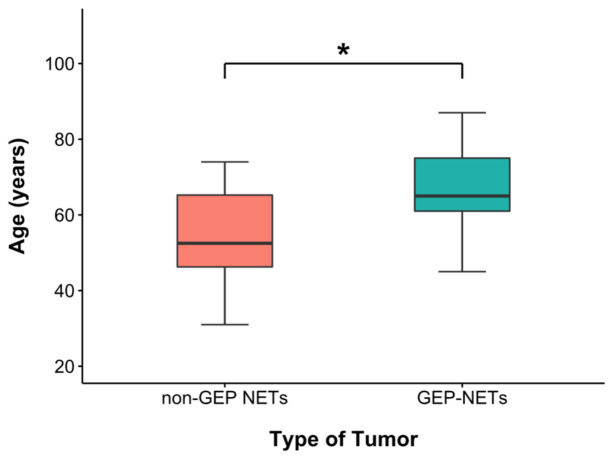
Box plot of current age (years) in patients with non-GEP NETs versus GEP-NETs. An independent samples *t*-test indicated a statistically significant between-group difference (*p* < 0.01). The box spans the interquartile range (25th–75th percentile) with the median shown as a horizontal line. Whiskers represent minimum and maximum values. * represents statistically significant difference (*p* < 0.05).

Conversely, independent samples *t*-tests and Mann–Whitney U tests yielded no statistically significant differences between G1 and G2 patients regarding all other evaluated continuous variables (all *p* > 0.05). When comparing participants based on tumor site classification, significant differences were observed regarding age-related parameters. Specifically, an independent samples *t*-test revealed that patients diagnosed with GEP-NETs were significantly older than those with non-GEP NETs (66.64 ± 11.01 years vs. 53.68 ± 12.49 years; t(59) = −4.206, *p* < 0.01, pFDR < 0.01, Cohen’s d = 1.10) (Figure 2).

**Figure 3:**
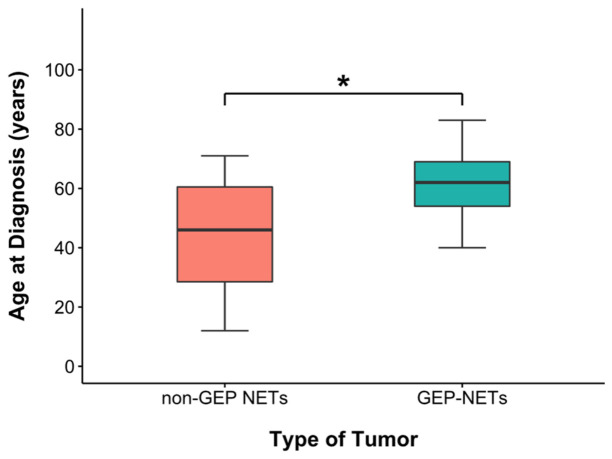
Box plot of age at diagnosis (years) in patients with non-GEP NETs versus GEP-NETs. A non-parametric Mann–Whitney U test indicated a statistically significant between-group difference (*p* < 0.01). The box spans the interquartile range (25th–75th percentile) with the median shown as a horizontal line. Whiskers represent minimum and maximum values. * represents statistically significant difference (*p* < 0.05).

Similarly, a non-parametric Mann–Whitney U test indicated a highly significant difference in the age at diagnosis between patients with non-GEP NETs and those with GEP-NETs [46.00 (IQR 32.00) years vs. 62.00 (IQR 15.00) years, U = 163, *p* < 0.01, pFDR ≤ 0.01, r = 0.52] (Figure 3).

Conversely, no statistically significant differences were observed between GEP-NET and non-GEP-NET patients regarding all other evaluated behavioral and dietary parameters, including ORTO-15, PREDIMED, and PAL scores (all *p* > 0.05). The ORTO-15 and PREDIMED survey scores are presented in Table 2.

**Figure 4:**
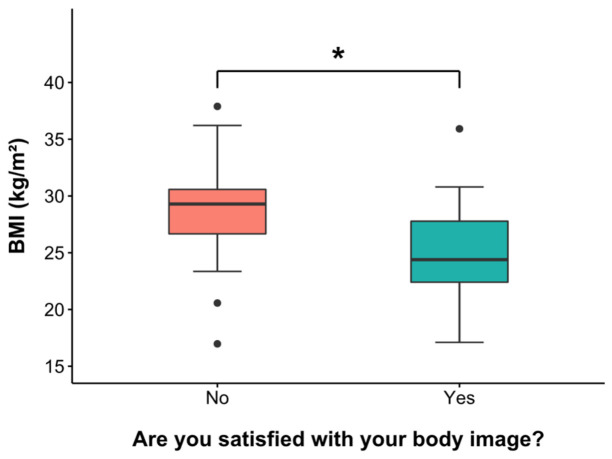
Box plot of BMI (kg/m^2^) in patients Dissatisfied vs. Satisfied with their body image. An independent samples *t*-test indicated a statistically significant between-group difference (*p* < 0.01). The box spans the interquartile range (25th–75th percentile) with the median shown as a horizontal line. Whiskers represent minimum and maximum values, while individual black dots represent outlier data points. * represents statistically significant difference (*p* < 0.05).

Based on the categorical analysis of the ORTO-15 scores, the prevalence of patients at risk of orthorexia was compared under two different literature-established cutoff values. Using the classic cutoff of <40 points, 88.5% (*n* = 54) of the sample was classified as at risk, whereas the more restrictive cutoff of <35 points identified 41.0% (*n* = 25) of the cohort as at risk. Regarding the ORTO-15 questionnaire, patients were stratified based on the cutoff threshold of <35 points to identify individuals at risk of orthorexia. An independent samples *t*-test revealed a statistically significant difference in age between patients at risk and those not at risk (58.00 ± 12.56 years vs. 64.72 ± 12.86 years; t(59) = 2.027, *p* = 0.047, pFDR = 0.078, Cohen’s d = 0.53), with younger patients exhibiting a higher risk. No statistically significant differences were observed for the other continuous variables (all *p* > 0.05). Furthermore, when patients were stratified using the traditional cutoff threshold of <40 points, no statistically significant differences were found between the two groups for any of the evaluated continuous variables (all *p* > 0.05). Based on the categorical analysis of the PREDIMED scores, 75.4% (*n* = 46) of the study sample exhibited moderate adherence to the MD. Furthermore, 18.0% (*n* = 11) showed low adherence, while only 6.6% (*n* = 4) met the criteria for high adherence. When asked about body image satisfaction, 67.2% (*n* = 41) of the participants reported being satisfied, whereas the remaining 32.8% (*n* = 20) expressed dissatisfaction. When comparing participants based on this parameter, an independent samples *t*-test revealed a significant difference in BMI between the two groups [28.86 ± 5.08 kg/m^2^ vs. 24.93 ± 3.93 kg/m^2^, t(59) = 3.329, *p* < 0.01, pFDR < 0.01, Cohen’s d = 0.91] (Figure 4).

**Figure 5:**
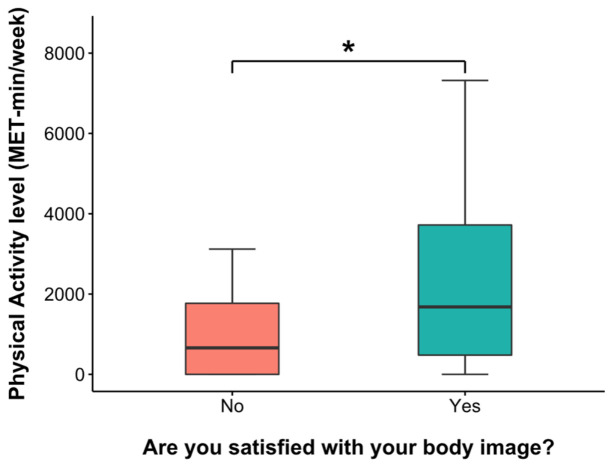
Box plot of physical activity level (MET-min/week) in patients dissatisfied vs. satisfied with their body image. A non-parametric Mann–Whitney U test indicated a statistically significant between-group difference (*p* = 0.021). The box spans the interquartile range (25th–75th percentile) with the median shown as a horizontal line. Whiskers represent minimum and maximum values. * represents statistically significant difference (*p* < 0.05).

Moreover, participants dissatisfied with their body image showed significantly lower PAL compared to the satisfied group [660.0 (IQR 1800.0) vs. 1680.0 (IQR 3240.0), U = 271, *p* = 0.021, pFDR = 0.042, r= 0.31] (Figure 5).

**Figure 6:**
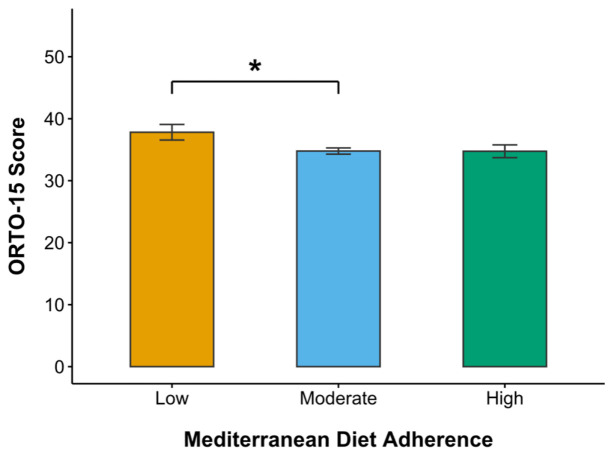
Bar plot of ORTO-15 total score across Mediterranean Diet Adherence categories (Low, Moderate, and High). A one-way ANOVA indicated a statistically significant main effect of adherence categories (*p* = 0.04), with post hoc comparisons showing a significant difference specifically between the low and moderate adherence groups (* *p* < 0.05). Bars represent the mean values, while error bars indicate the standard error of the mean (SEM).

All other analyzed variables, including ORTO-15 and PREDIMED scores, age, body mass, height, disease duration, and age at diagnosis, yielded no statistically significant differences between dissatisfied and satisfied (all *p* > 0.05). Regarding gender differences, no statistically significant differences were observed between male and female participants regarding all analyzed variables, including ORTO-15 and PREDIMED scores, BMI, PAL, height, current age, disease duration, and age at diagnosis (all *p* > 0.05). Regarding pharmacological management, direct comparisons between patients receiving SSA therapy and those who were untreated revealed no statistically significant differences across all evaluated parameters, including ORTO-15 and PREDIMED scores, weight, height, BMI, PAL, current age, disease duration, and age at diagnosis (all *p* > 0.05). A one-way ANOVA was conducted to examine the impact of marital status on the studied clinical and anthropometric variables. The analysis revealed a statistically significant main effect of marital status on body weight (F(4, 56) = 2.774, *p* = 0.03, eta-squared η^2^ = 0.165). Post hoc comparisons using Tukey’s HSD test indicated that separated individuals had a significantly lower body weight compared to both single/never-married participants (*p* = 0.03, Cohen’s d = −1.41) and married participants (*p* = 0.03, Cohen’s d = −1.48). No statistically significant differences in weight were observed among the other marital status groups. Furthermore, the ANOVA and Kruskal–Wallis tests (for non-parametric variables) yielded no significant differences across marital status categories for any of the other evaluated continuous variables (all *p* > 0.05). A one-way ANOVA was conducted to evaluate the effect of MD adherence (categorized as high, moderate, and low based on the PREDIMED score) on the ORTO-15 total score. The analysis revealed a statistically significant main effect of PREDIMED categories on the ORTO-15 score (F(2, 58) = 3.399, *p* = 0.04, eta-squared η^2^ = 0.105). Post hoc comparisons using Tukey’s HSD test indicated that participants with low MD adherence exhibited a significantly higher ORTO-15 score compared to those with moderate adherence [37.82 ± 4.17 vs. 34.78 ± 3.41, *p* = 0.032, pFDR = 0.078, Cohen’s d = 0.83] (Figure 6).

No statistically significant differences were observed between the high adherence group and the other categories. Furthermore, the ANOVA and Kruskal–Wallis tests (for non-parametric variables) yielded no significant differences across PREDIMED categories for any of the other evaluated continuous variables, including BMI, PAL, age, and disease duration (all *p* > 0.05). Regarding educational level, ANOVA and Kruskal–Wallis tests revealed no statistically significant differences across the different educational categories for any of the evaluated continuous variables, including anthropometric, clinical, and behavioural parameters (all *p* > 0.05). A correlation analysis was conducted to explore potential relationships among the continuous variables, which included demographic and clinical characteristics (current age, age at diagnosis, disease duration), anthropometric parameters (weight, height, BMI), PAL, ORTO-15 and PREDIMED. The analysis revealed no statistically significant correlations between any of the evaluated parameters (all *p* > 0.05).

### Multivariate Regression Models

In the overall cohort (*n* = 61), logistic regression on body image dissatisfaction (Model 1; Likelihood Ratio test *p* = 0.0038) showed that both BMI (OR = 1.222, 95% CI: 1.051 to 1.422, *p* = 0.009) and physical activity levels (PAL, OR = 0.615 per 1000 MET-min/week, 95% CI: 0.390 to 0.971, *p* = 0.037) were significant independent predictors of body image dissatisfaction, whereas age, gender, and GEP site did not show significant associations. In the graded subgroup (*n* = 40), logistic regression (Model 1b; Likelihood Ratio test *p* = 0.0017) confirmed that BMI (OR = 1.292, 95% CI: 1.020 to 1.636, *p* = 0.033) and physical activity (OR = 0.409 per 1000 MET-min/week, 95% CI: 0.187 to 0.893, *p* = 0.023) remained independently associated with body image dissatisfaction, while tumor grading (G2 vs. G1) was not significant (OR = 0.184, *p* = 0.137). Linear regression models adjusting for covariates showed that PREDIMED score was not an independent predictor of ORTO-15 continuous score (Model 2; beta = −0.433, *p* = 0.172). Finally, in the grading subgroup, multiple linear regression for PREDIMED score (Model 3) showed that physical activity was independently associated with dietary adherence (beta = 0.335, *p* = 0.023), but the difference between G1 and G2 grading levels was no longer significant after adjusting for all covariates (beta = −0.856, *p* = 0.113) (Table 3).

## 4. Discussion

Our findings reveal three noteworthy behavioral patterns in NET survivors: a significant association between body image dissatisfaction and both higher BMI and lower physical activity; a link between moderate MD adherence and orthorexic tendencies; and the absence of any detrimental effect of SSA therapy on behavioral parameters.

Patients who were dissatisfied with their body image exhibited a significantly higher BMI and markedly reduced PAL compared to those who reported being satisfied. This clear divergence between the two groups sheds light on the functional and psychological burden of NETs. In our cohort, dissatisfied patients presented a mean BMI indicative of overweight (28.86 kg/m^2^), whereas satisfied patients fell within the normal weight range (24.93 kg/m^2^). Crucially, this dissatisfaction was mirrored by a drastic reduction in PAL (660.0 vs. 1680.0 MET-min/week). These findings suggest the existence of a potential “vicious cycle” in NET survivorship: disease- or treatment-related changes in body composition may trigger body image dissatisfaction, which subsequently acts as a powerful psychological barrier to engaging in physical activity, thereby further exacerbating weight gain and functional impairment. This proposed pathological loop aligns with the recent literature documented in other oncological populations [33,34,35,36]. Our multivariate regression analyses further enhance the persuasiveness of these conclusions. Even after adjusting for key confounders such as age, gender, and tumor characteristics, both BMI (OR = 1.222, *p* = 0.009) and physical activity levels (PAL, OR = 0.615 per 1000 METs, *p* = 0.037) remained independently and significantly associated with body image dissatisfaction. This reinforces the clinical relevance of our proposed “vicious cycle” in NET survivorship: body weight and physical functioning are main determinants of body image perception, and their relationship holds true independently of clinical tumor features.

Regarding the ORTO-15 questionnaire, our participants achieved a mean score of 35.3 ± 3.6. Traditionally, a diagnostic cut-off score of <40 was established to identify orthorexic tendencies [29]. However, this threshold has been widely criticized for generating an excessively high rate of false positives, capturing simple healthy eating habits rather than true pathology [31]. Consequently, the recent literature advocates for a more restrictive cut-off of <35 to increase diagnostic precision [30,31,32]. When evaluated against this stricter modern criterion, our patients’ mean score demonstrates that, on average, they do not present manifest orthorexic behaviors. Nevertheless, this score sits close to the modern limit, suggesting that a substantial proportion of the cohort is positioned near the clinical risk threshold. A highly novel aspect of our study is the exploration of ON in relation to diet adherence among NET patients. Interestingly, patients with moderate adherence to the MD recorded significantly lower ORTO-15 scores (34.78) compared to those with low adherence (37.82). Given that a lower score on the ORTO-15 questionnaire reflects a higher risk of ON, our data suggest that patients who actively attempt to follow a structured, “healthy” dietary pattern, like the MD, may be more prone to developing obsessive or rigid eating behaviors. However, our multivariate linear regression analysis (Model 2) demonstrated that Mediterranean Diet adherence is not a significant independent predictor of ORTO-15 scores when adjusting for covariates (beta = −0.433, *p* = 0.172), and the association did not remain significant after FDR correction (pFDR = 0.078). Therefore, our findings do not support a progressive, linear dose–response relationship, indicating that this link is exploratory rather than a progressive pathological continuum. A cancer diagnosis frequently acts as a catalyst for dietary changes, as patients seek to regain a sense of control over their health [13]. However, the fear of disease recurrence and the chronic psychological burden of the pathology can inadvertently push these health-conscious adjustments toward maladaptive extremes, transforming a balanced diet into an overly restrictive and rigid eating behaviour [14,15,16,17]. The comparison of ORTO-15 cutoff values also yields important clinical insights. While the classic cutoff of <40 points classified the vast majority of our patients (88.5%) as at risk of orthorexia, it failed to show any statistically significant correlation with clinical or nutritional parameters, suggesting a high rate of false positives. In contrast, the stricter cutoff of <35 points identified a more specific subpopulation (41.0% at risk). Crucially, the <35 cutoff was able to reveal that patients at risk of orthorexia were significantly younger (58.00 ± 12.56 years) than those not at risk (64.72 ± 12.86 years, *p* = 0.047), a finding that is fully consistent with general population and oncological data showing higher vulnerability to eating rigidity in younger cohorts. This age difference was completely diluted and lost when using the <40 threshold (*p* = 0.600), further justifying the clinical utility of the stricter < 35 cutoff. However, single-scale assessments have inherent limitations, and clinical behavioral observations should ideally be combined in clinical practice to confirm a diagnosis.

Regarding dietary habits, the cohort achieved a median PREDIMED score of 7.0, with an interquartile range of 2.0. Looking at the adherence classes, the frequency distribution reveals that the vast majority of participants, specifically 75.4% (*n* = 46), showed moderate adherence to the MD. Conversely, 18.0% (*n* = 11) demonstrated low adherence, while only a small minority of 6.6% (*n* = 4) met the criteria for high adherence. This prominent clustering within the moderate adherence class suggests that while patients with NETs maintain a baseline awareness of healthy dietary habits, there remains a substantial margin for clinical improvement and targeted nutritional counselling. In oncology, a high adherence to the MD is strongly associated with anti-inflammatory benefits, better treatment tolerance, and improved overall quality of life [12]. Therefore, this widespread moderate adherence represents a positive baseline starting point. However, there is still room for improvement, and clinical nutritionists could play a key role in safely guiding these patients toward a higher dietary quality. This nutritional support must be managed carefully to ensure that encouraging healthier habits does not lead to obsessive or overly restrictive behaviors, which could ultimately trigger eating disorders. Crucially, clinicians must avoid pathologizing adaptive, health-promoting eating behaviors in cancer survivors who are actively engaging in self-care. Patients who have survived a rare tumor like a NET often monitor their diet closely as a rational and positive coping strategy to manage gastrointestinal symptoms and improve their overall health [9,13]. This vigilance represents a healthy, adaptive self-care behavior (dietary monitoring) rather than a clinical eating disorder, and the moderate shift in ORTO-15 scores across the cutoff should be interpreted as a potential risk of rigidity rather than a pathological condition [10,11]. Although the cross-sectional nature of our study does not allow us to establish definitive clinical nutritional recommendations, our findings suggest a preliminary stratification with a view to future interventions. For example, patients with poor adherence to the Mediterranean diet and a negative body image might benefit from integrated psycho-nutritional counselling; conversely, those with high dietary adherence but a high risk of orthorexia (ORTO-15 < 35) might require non-restrictive approaches to prevent eating disorders. These observation-based, personalised profiles require validation through future prospective and interventional studies.

With regard to the PAL, our cohort exhibited an active lifestyle [28]. In particular, physical activity levels calculated using the GPAQ revealed a consistent median value of 1080.0 MET-min/week, demonstrating that the average patient remains significantly active and physically functional [28]. Furthermore, even patients who are dissatisfied with their body image, compared to those who are satisfied, despite having significantly lower levels of physical activity, still reach the METs required to be considered physically active [28].

With regard to the clinical and histological classification of our sample, the majority of the cohort was diagnosed with GEP-NETs, 63.9%, while the remaining 36.1% were diagnosed with non-GEP NETs. Furthermore, the grading distribution among GEP-NET patients showed a high prevalence of well-differentiated, low-grade tumors, with 61.0% classified as G1 and 36.6% as G2, compared to only a very small minority of high-grade G3 lesions (2.4%). This clinical distribution is highly representative of the typical outpatient NETs population, where indolent or slow-growing tumors predominate [37,38]. When examining the impact of tumor grading on behavioral parameters, our analysis revealed a statistically significant difference in dietary habits, with G1 patients showing significantly higher adherence to the MD compared to those with G2 lesions. This finding is of considerable clinical interest. Patients diagnosed with G2 tumors, which are characterized by an intermediate grade and a potentially more aggressive clinical course compared to the indolent G1, may experience higher levels of psychological distress and anxiety regarding their prognosis [25,26]. This increased psychological burden could inadvertently lead to a loss of motivation in maintaining a strict, healthy dietary pattern, potentially triggering mechanisms of emotional eating or dietary neglect [14,15,16,17,25]. Furthermore, a higher tumor grade might be associated with slightly more pronounced clinical symptoms or the need for more intensive therapeutic management [24,39], both of which might create functional barriers to following a structured, high-quality nutritional regimen such as the MD [40]. Consequently, while initial observations suggested poorer dietary adherence in G2 patients, our multivariate analysis revealed that tumor grading itself does not have an independent effect on dietary habits. Instead, G2 patients exhibited lower adherence primarily because of their reduced levels of physical activity. Therefore, individualized interventions for this subgroup should go beyond isolated nutritional counselling. In line with the recent literature [41,42], implementing tailored physical exercise programs could represent a crucial strategy not only to enhance their overall psychophysical well-being but also to indirectly improve and sustain their adherence to the Mediterranean diet, as previously observed in other oncological populations [43,44].

Regarding pharmacological management, data on SSA therapy showed that 45.6% (*n* = 26) of patients were currently receiving the treatment. Crucially, our sub-group analysis revealed that variables including ORTO-15, PREDIMED, BMI, and PAL did not differ significantly based on treatment status. From a clinical perspective, this lack of statistically significant differences represents a highly relevant finding for neuroendocrine oncology. SSAs are lifelong first-line therapies that, despite their effectiveness in controlling tumor growth and secretory syndromes [40,45], are frequently associated with gastrointestinal side effects such as steatorrhea, abdominal bloating, and altered nutrient absorption [40]. These symptoms could theoretically force patients to implement restrictive dietary modifications or reduce their physical activity due to abdominal discomfort [40]. However, our data demonstrate that patients undergoing SSA therapy maintain the same physical activity levels and MD adherence as their untreated peers, without showing any increased risk of rigid or orthorexic eating behaviors. This suggests that modern pharmacological management for NETs is well-tolerated and does not act as a direct behavioral deterrent, allowing patients to safely pursue active and healthy lifestyles during their survivorship pathway. Furthermore, while SSA therapy was generally well-tolerated in our cohort, its potential gastrointestinal side effects (e.g., steatorrhea, diarrhea, and abdominal bloating) necessitate specific clinical nursing and lifestyle adaptations. From a nutritional perspective, patients experiencing SSA-induced gastrointestinal distress should receive individualized counselling to temporarily adapt their MD; this includes consuming smaller, more frequent meals, managing dietary fat intake, favoring easily digestible soluble fibers, and evaluating the need for Pancreatic Enzyme Replacement Therapy (PERT) when clinically indicated. From a physical activity standpoint, nursing protocols should encourage low-to-moderate intensity exercises (e.g., walking or mindful movement practices) tailored to patient tolerance. These activities can promote bowel regularity and reduce bloating without exacerbating treatment-related fatigue or gastrointestinal hypermotility. A crucial clinical finding emerging from our study is the statistically significant association between tumor site classification and pharmacological management. Specifically, our analysis revealed that patients diagnosed with GEP-NETs had an almost fourfold higher likelihood of being treated with SSA compared to those presenting with non-GEP NETs. From an oncological perspective, this pronounced therapeutic asymmetry is entirely consistent with international guidelines and standard clinical practice [24,39,46]. SSA, like octreotide and lanreotide, represent the undisputed first-line systemic therapy for GEP-NETs due to their dual mechanism of action [24,39]. Therefore, the higher prevalence of SSA therapy among GEP-NET patients in our cohort (56.8% vs. 25.0%) directly reflects standard-of-care medical stratification rather than an artifact of our sample. Crucially, while this indicates that a substantial portion of our GEP-NET subgroup was exposed to the chronic, potential gastrointestinal side effects typical of SSA [47], it underscores the relevance of our broader behavioral findings: the lack of differences in lifestyle parameters between treated and untreated subjects proves that this necessary clinical polarization does not translationally impair the patients’ ability to adhere to healthy diets or maintain active physical routines during their survivorship.

Another highly relevant clinical finding emerging from our study is the pronounced demographic divergence between the tumor site subgroups, with patients diagnosed with GEP-NETs being significantly older, both currently and at the time of their initial diagnosis, compared to those presenting with non-GEP NETs. From an epidemiological perspective, this age gap is fully consistent with current global registry data and the known natural history of neuroendocrine malignancies [48,49]. GEP-NETs typically peak in incidence between the sixth and seventh decades of life [48,49]. Because many GEP-NETs (particularly those originating in the stomach, small intestine, or pancreas) are slow-growing and non-functioning, they often exhibit a silent or indolent clinical progression [48,49]. This lack of early, specific symptoms frequently delays clinical detection until the disease is discovered incidentally during routine screenings, endoscopic procedures, or imaging studies performed for unrelated abdominal complaints, leading to a higher age at diagnosis [48,49]. In contrast, non-GEP NETs, which in our cohort predominantly encompass thoracic, bronchial, or other extra-gastrointestinal sites, frequently manifest at an earlier age [50,51]. This clinical presentation is often driven by the fact that thoracic or bronchial NETs tend to cause immediate mechanical or respiratory symptoms (such as persistent cough, recurrent hemoptysis, or localized airway obstruction) even when the primary lesion is relatively small [50,51]. These acute clinical signs prompt earlier diagnostic investigations, resulting in a significantly younger age at diagnosis [50,51]. Based on these clinical observations, while the cross-sectional nature of our study precludes the establishment of definitive clinical guidelines, our findings can hypothesize a preliminary stratification for future interventions. For instance, patients with low Mediterranean diet adherence and poor body image might benefit from integrated psycho-nutritional counselling; conversely, those with high dietary adherence but a high orthorexia risk (ORTO-15 < 35) might require non-restrictive approaches to prevent disordered eating. These tailored observation-based profiles require validation through future prospective and interventional trials.

### Limitations

Despite the clinical relevance of our findings, several study limitations must be addressed. First, the cross-sectional design of this research inherently precludes the establishment of definitive causal relationships between tumor clinical parameters and the evaluated behavioral or psychometric outcomes. Second, the study lacked a healthy, age- and gender-matched control group or a comparative cohort of patients with other tumor types, which limits the ability to determine whether these specific lifestyle and psychometric patterns are uniquely characteristic of NET survivors or merely reflective of the general population or other oncological cohorts. Third, the sample size (*n* = 61) is relatively small and patients were recruited from a single clinical center, which limits the generalizability and extrapolation of the conclusions to the entire heterogeneous NET population.

Fourth, anthropometric evaluations were limited exclusively to BMI calculated from weight and height. Although BMI is a universally accepted clinical metric, it cannot differentiate between fat mass and lean muscle tissue. Incorporating comprehensive body composition analysis, such as Bioelectrical Impedance Analysis or Dual-Energy X-ray Absorptiometry, would have provided critical data on muscle mass quality and sarcopenia, which are highly relevant parameters in oncological populations. Fifth, body image satisfaction was evaluated using a single self-reported dichotomous question. While this single-item approach was pragmatically selected to minimize patient burden and reduce administration time during routine outpatient clinical visits, we acknowledge that it represents a methodological limitation, as it cannot capture the multidimensional psychological complexity of body image disturbances as effectively as comprehensive, validated multi-item psychometric scales. Sixth, our study exclusively enrolled outpatients and did not include hospitalized patients or those in advanced, end-stage disease. This introduces a selection bias, as outpatients are generally more functional and medically stable. Seventh, all behavioral and dietary data were collected via self-report questionnaires, including the GPAQ, PREDIMED, and ORTO-15, and no actual nutritional or dietary intake measurements were taken. Specifically, physical activity levels assessed via the GPAQ should be interpreted with caution, as self-reported questionnaires are prone to recall bias and tend to overestimate actual daily activity. With specific regard to the ORTO-15, although it is one of the most widely used screening tools in the literature, its internal consistency (alpha = 0.643 in our sample) and factor instability are the subject of extensive debate [10,31], which often results in a high rate of false positives that requires cautious clinical interpretation. Eighth, the lack of an a priori power calculation increases the risk of Type II errors for weaker associations or small subgroup differences (with a minimum detectable correlation of r = 0.35 at 80% power), meaning that findings should be interpreted as exploratory.

Finally, critical clinical variables that could significantly influence eating behaviors and body image, such as the presence of metastases, gastrointestinal symptoms, and undiagnosed depression, were not evaluated.

## 5. Conclusions

This study provides novel insights into the lifestyle and psychometric profile of NET survivors. Most patients maintain adequate physical activity levels and moderate MD adherence; however, body image dissatisfaction—present in nearly one-third of the cohort—is significantly associated with higher BMI and markedly reduced physical activity, suggesting a potentially self-reinforcing cycle. Although moderate dietary adherence was associated with orthorexic risk in simple comparisons, this link did not remain significant after FDR correction or in multivariate models, suggesting that orthorexic tendencies in this cohort should be interpreted as preliminary and exploratory, without demonstrating a progressive dose–response gradient. Furthermore, physical activity level, rather than tumor grading, emerges as the primary independent driver of dietary adherence. Consequently, the lower dietary adherence observed in G2 patients appears to be driven by their reduced physical activity; therefore, targeted interventions aimed at increasing daily physical activity and implementing structured exercise programs in this vulnerable subgroup could effectively improve and sustain their adherence to the Mediterranean diet. Notably, SSA therapy does not adversely affect behavioral parameters, confirming its tolerability in the survivorship setting. These findings support the integration of nutritional counselling, physical activity promotion, and psychological support into multidisciplinary NET care, targeting modifiable factors that may influence long-term outcomes and quality of life.

## Figures and Tables

**Table 1:** Characteristics of participants.

Characteristics	
Age (years)	62.0 ± 13.1
Body Mass (kg)	73.2 ± 14.8
Height (m)	1.67 ± 0.09
BMI (kg/m^2^)	26.2 ± 4.7
Duration of NET (years)	3.5 (6.0)
Diagnosis age (years)	59.0 (19.0)
PAL (MET-min/week)	1080.0 (2640.0)

Data are expressed as mean ± SD and median (IQR). BMI = Body Mass Index, PAL = Physical Activity Levels.

**Table 2:** ORTO-15 and PREDIMED survey scores.

Survey	Score
ORTO-15	35.3 ± 3.6
PREDIMED	7.0 (2.0)

Data are expressed as mean ± SD and median (IQR).

**Table 3:** Multivariate Regression Models For Body Image Dissatisfaction and Dietary Adherence/Orthorexia.

Model/Dependent Variable	Predictor	Coef/Beta	SE	OR (95% CI)	*p*
Model 1: Body Image Dissatisfaction (Overall Cohort, *n* = 61) Logit Model (LLR *p* = 0.0038)	BMI (kg/m^2^)	0.201	0.077	1.222 (1.051–1.422)	**0.009**
	PAL (per 1000 METs)	−0.486	0.233	0.615 (0.390–0.971)	**0.037**
	Age (years)	−0.014	0.029	0.99 (0.93–1.05)	0.635
	Gender (0 = F, 1 = M)	−0.743	0.642	0.48 (0.14–1.68)	0.248
	GEP (0 = No, 1 = Yes)	0.106	0.788	1.11 (0.24–5.22)	0.893
Model 1b: Body Image Dissatisfaction (Graded Subgroup, *n* = 40) Logit Model (LLR *p* = 0.0017)	BMI (kg/m^2^)	0.256	0.120	1.292 (1.020–1.636)	**0.033**
	PAL (per 1000 METs)	−0.893	0.394	0.409 (0.187–0.893)	**0.023**
	Age (years)	−0.105	0.057	0.90 (0.81–1.01)	0.065
	Gender (0 = F, 1 = M)	−1.719	1.003	0.18 (0.03–1.28)	0.087
	Grade G2 (0 = G1, 1 = G2)	−1.693	1.139	0.184 (0.022–1.724)	0.137
Model 2: ORTO-15 Score (Overall Cohort, *n* = 61) Linear Model (R^2^ = 0.068)	PREDIMED Score	−0.433	0.313	N/A	0.172
	BMI (kg/m^2^)	0.070	0.105	N/A	0.507
	PAL (per 1000 METs)	0.046	0.264	N/A	0.861
	Age (years)	0.032	0.043	N/A	0.457
	Gender (0 = F, 1 = M)	−0.178	0.957	N/A	0.853
	GEP (0 = No, 1 = Yes)	0.565	1.147	N/A	0.624
Model 3: PREDIMED Score (Graded Subgroup, *n* = 40) Linear Model (R^2^ = 0.242)	Grade G2 (0 = G1, 1 = G2)	−0.856	0.526	N/A	0.113
	BMI (kg/m^2^)	−0.000	0.054	N/A	0.994
	PAL (per 1000 METs)	0.335	0.141	N/A	**0.023**
	Age (years)	0.020	0.024	N/A	0.422
	Gender (0 = F, 1 = M)	0.016	0.493	N/A	0.974

Model 1 and Model 1b are multivariate logistic regression models; Coef represents the log-odds regression coefficient, and effect sizes are reported as Odds Ratios (OR) with 95% confidence intervals (CI). Model 2 and Model 3 are multiple linear regression models; Coef represents the unstandardized Beta weight (beta). Intercepts for all models are omitted to focus on clinical predictors. BMI = Body Mass Index; PAL = Physical Activity Level (scaled per 1000 MET-min/week, where 1 unit represents 1000 MET-min/week); GEP = Gastroenteropancreatic tumor site; LLR = Likelihood Ratio; SE = Standard Error; N/A = Not Applicable. Bold face type indicates statistically significant predictors (*p* < 0.05).

## Data Availability

The data that support the findings of this study are available from the corresponding author upon reasonable request due to privacy and ethical restrictions (to protect patient confidentiality).

## Abbreviations

NENs	Neuroendocrine Neoplasms
NETs	Neuroendocrine Tumors
GEP	Gastroenteropancreatic
GEP-NETs	Gastroenteropancreatic Neuroendocrine Tumors
NECs	Neuroendocrine Carcinomas
WHO	World Health Organization
MD	Mediterranean Diet
ON	Orthorexia Nervosa
BMI	Body Mass Index
GPAQ	Global Physical Activity Questionnaire
MET	Metabolic Equivalent
PAL	Physical Activity Levels
SD	Standard Deviation
OR	Odds Ratio
IQR	Interquartile Ranges
SSA	Somatostatin Analog
FDR	False Discovery Rate
CI	Confidence Interval
PERT	Pancreatic Enzyme Replacement Therapy
